# Supplementary material for: A Bacillus thuringiensis Cry protein controls soybean cyst nematode in transgenic soybean plants
Source: Nat Commun. 2021 Jun 7;12:3380. doi: 10.1038/s41467-021-23743-3 (PMC8184815; doi:10.1038/s41467-021-23743-3)
Supplement: Supplementary file 2 — Supplementary Information [file 41467_2021_23743_MOESM2_ESM.pdf]

A *Bacillus thuringiensis* Cry protein controls soybean cyst nematode in transgenic soybean plants

Kahn *et al.*

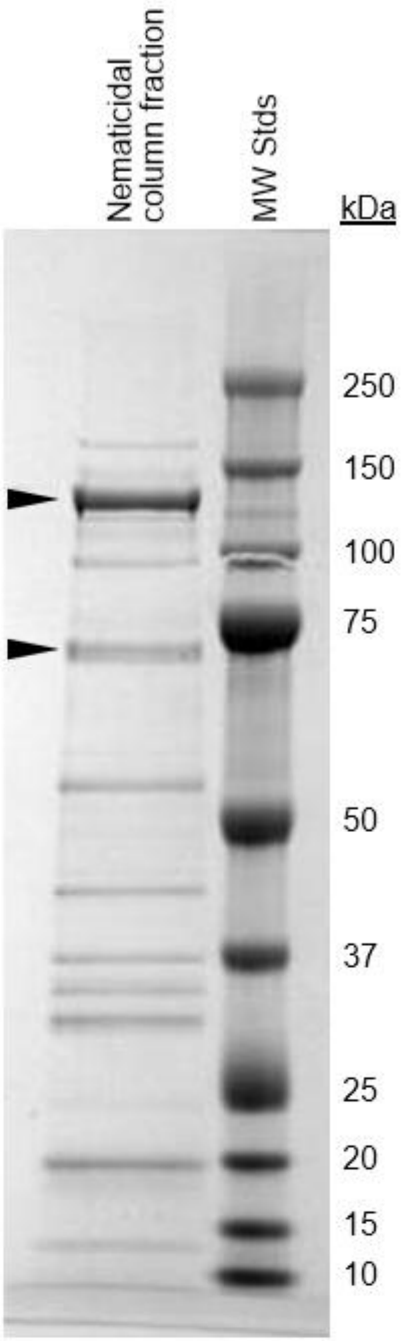

Supplementary Figure 1. Proteins present in an active fraction from a *Bacillus thuringiensis* strain. An aliquot from a column fraction with strong nematicidal activity against *Caenorhabditis elegans* was run on SDS-PAGE, blotted to PVDF, and stained with Coomassie, and protein bands were cut out and subjected to N-terminal sequencing. The N-terminus of the marked bands at about 130 kDa and 70 kDa were consistent with the N-terminus of Cry14Aa. The fractionation and N-terminal sequencing were done twice, and gave similar results both times.

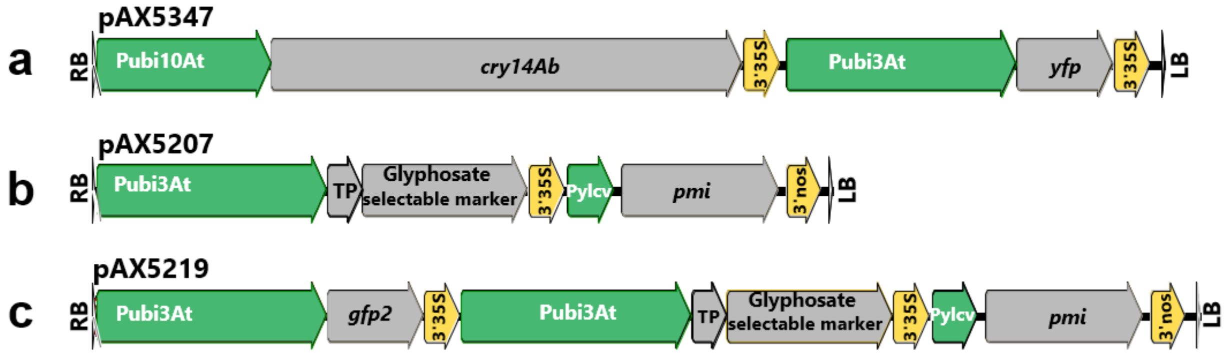

Supplementary Figure 2. Diagram of T-DNA for expression of Cry14Ab in soybean. RB: right border of T-DNA[1]; LB: left border of T-DNA[1]; Pubi10At: *Arabidopsis thaliana* ubiquitin-10 gene promoter[2]; 3’ 35S: 3' untranslated region of the 35S transcript of cauliflower mosaic virus[3]; *yfp*: yellow fluorescent protein gene[4]; TP: chloroplast transit peptide[5]; Pylcv: promoter for the transcript of *Cestrum* yellow leaf curling virus[6]; *pmi*: phosphomannose isomerase gene[7]; 3’ nos: 3’ untranslated region of *Agrobacterium tumefaciens* nopaline synthase gene[8]; *gfp2*: green fluorescent protein 2 gene[9]. a) pAX5347 contains a nematicidal gene cassette and a *yfp* transformation marker cassette, and was used for co-transformation with pAX5207 to produce plants expressing Cry14Ab. b) pAX5207 contains a glyphosate selectable marker cassette and a *pmi* cassette, and was used for co-transformation with pAX5347. c) pAX5219 contains a glyphosate selectable marker cassette and a *gfp2* cassette, and was used alone for transformation to produce negative control plants.

Supplementary Table 1. Primers used in this study.

Name	Identity	Sequence
050324C	Cry14Ab ORF 5' start	GGGGACAAGTTTGTACAAAAAAGCAGGCTTCAAAGGAGGTGATTAAAATGGATTGTAATTTACAATCACAAC
050324B	Cry14Ab ORF 3' end	GGGGACCACTTTGTACAAGAAAGCTGGGTCTTATTCGTCGTCTGATAATTCAA
Cry14Ab_p2f	Cry14Ab fragment forward primer	GAA CGG AAA CTT CGA TGT TG
Cry14Ab_p3r	Cry14Ab fragment reverse primer	CAT CGA GGT ACT TAG AGT TC

Supplementary references

1. Zambryski, P. Basic processes underlying *Agrobacterium*-mediated DNA transfer to plant cells. *Annual Review of Genetics* **22**, 1–30 (1988).
2. Grefen, C. *et al.* A ubiquitin-10 promoter-based vector set for fluorescent protein tagging facilitates temporal stability and native protein distribution in transient and stable expression studies. *Plant Journal* **64**, 355–365 (2010).
3. Sanfaçon, H., Brodmann, P. & Hohn, T. A dissection of the cauliflower mosaic virus polyadenylation signal. *Genes & Development* **5**, 141-149 (1991).
4. yfp - Yellow fluorescent protein - *Aequorea victoria* (Jellyfish) - yfp gene & protein. Available at: https://www.uniprot.org/uniprot/A0A059PIR9.
5. *Glycine max* cDNA, clone: GMFL01-45-K13. (2008). GenBank accession AK245902.
6. Stavolone, L., Ragozzino, A. & Hohn, T. Characterization of *Cestrum* yellow leaf curling virus: a new member of the family Caulimoviridae. *Journal of General Virology* **84**, 3459-3464 (2003).
7. He, Z. *et al.* Phosphomannose-isomerase (*pmi*) gene as a selectable marker for rice transformation via Agrobacterium. *Plant Science* **166**, 17–22 (2004).
8. Depicker, A., Stachel, S., Dhaese, P., Zambryski, P. & Goodman, H. Nopaline synthase: transcript mapping and DNA sequence. *Journal of Molecular and Applied Genetics* **1**, 561-573 (1982).
9. GFP2 - Green fluorescent protein 2 - *Astrangia haimei* (Cup coral) - GFP2 gene & protein. Available at: https://www.uniprot.org/uniprot/Q6R8F4.
