# Supplementary material for: A Bacillus thuringiensis Cry protein controls soybean cyst nematode in transgenic soybean plants
Source: Nat Commun. 2021 Jun 7;12:3380. doi: 10.1038/s41467-021-23743-3 (PMC8184815; doi:10.1038/s41467-021-23743-3)
Supplement: Supplementary file 3 — Reporting Summary [file 41467_2021_23743_MOESM3_ESM.pdf]

Corresponding author(s): Theodore W. Kahn

Last updated by author(s): Apr 24, 2021

Reporting Summary

Nature Research wishes to improve the reproducibility of the work that we publish. This form provides structure for consistency and transparency in reporting. For further information on Nature Research policies, see our Editorial Policies and the Editorial Policy Checklist.

Statistics

For all statistical analyses, confirm that the following items are present in the figure legend, table legend, main text, or Methods section.

n/a	Confirmed	
☐	☒	The exact sample size (*n*) for each experimental group/condition, given as a discrete number and unit of measurement
☐	☒	A statement on whether measurements were taken from distinct samples or whether the same sample was measured repeatedly
☐	☒	The statistical test(s) used AND whether they are one- or two-sided<br>*Only common tests should be described solely by name; describe more complex techniques in the Methods section.*
☒	☐	A description of all covariates tested
☒	☐	A description of any assumptions or corrections, such as tests of normality and adjustment for multiple comparisons
☐	☒	A full description of the statistical parameters including central tendency (e.g. means) or other basic estimates (e.g. regression coefficient) AND variation (e.g. standard deviation) or associated estimates of uncertainty (e.g. confidence intervals)
☐	☒	For null hypothesis testing, the test statistic (e.g. *F*, *t*, *r*) with confidence intervals, effect sizes, degrees of freedom and *P* value noted<br>*Give P values as exact values whenever suitable.*
☒	☐	For Bayesian analysis, information on the choice of priors and Markov chain Monte Carlo settings
☒	☐	For hierarchical and complex designs, identification of the appropriate level for tests and full reporting of outcomes
☒	☐	Estimates of effect sizes (e.g. Cohen's *d*, Pearson's *r*), indicating how they were calculated

Our web collection on statistics for biologists contains articles on many of the points above.

Software and code

Policy information about availability of computer code

Data collection	No software was used.
Data analysis	Microsoft Excel, Version 2008.<br>MLA "Quest Graph™ LC50 Calculator." AAT Bioquest, Inc, 24 Jul. 2020, https://www.aatbio.com/tools/lc50-calculator.<br>The R Project for Statistical Computing (https://www.r-project.org/), version 3.5.2.<br>Biorad Image Lab, version 6.0.0.<br>Microsoft Excel, version 2008.<br>TIBCO Spotfire, version 7.10.1.

For manuscripts utilizing custom algorithms or software that are central to the research but not yet described in published literature, software must be made available to editors and reviewers. We strongly encourage code deposition in a community repository (e.g. GitHub). See the Nature Research guidelines for submitting code & software for further information.

Data

Policy information about availability of data

All manuscripts must include a data availability statement. This statement should provide the following information, where applicable:

- Accession codes, unique identifiers, or web links for publicly available datasets
- A list of figures that have associated raw data
- A description of any restrictions on data availability

The source data that support the findings of this study are provided with this paper, and are available in figshare with the identifier

Cry14Ab is GenBank Accession AAA21516 (https://www.ncbi.nlm.nih.gov/protein/AAA21516).
RefSeq, the NCBI Reference Sequence Database, is available at https://www.ncbi.nlm.nih.gov/refseq/.

Field-specific reporting

Please select the one below that is the best fit for your research. If you are not sure, read the appropriate sections before making your selection.

☒ Life sciences ☐ Behavioural & social sciences ☐ Ecological, evolutionary & environmental sciences

For a reference copy of the document with all sections, see nature.com/documents/nr-reporting-summary-flat.pdf

Life sciences study design

All studies must disclose on these points even when the disclosure is negative.

Sample size	Sample size in the soybean field trial was determined by seed availability. Four replications were utilized in the 2010 field season due to seed limitations, with unequal replication of treatments with additional available seed. Eight replications with equal replication of all treatments were utilized in 2011 as additional seed was available.
Data exclusions	No data were excluded from the soybean field experiment.
Replication	Soybean field trial treatments were replicated within a year in a randomized complete block design. The experiment was also replicated with two years of data (2010 and 2011). These were the only attempts at replication, and the replication was successful.
Randomization	The soybean field experiment utilized a randomized complete block design.
Blinding	Soil samples from the soybean field trial were processed by technicians provided plot numbers, but not treatment assignments.

Reporting for specific materials, systems and methods

We require information from authors about some types of materials, experimental systems and methods used in many studies. Here, indicate whether each material, system or method listed is relevant to your study. If you are not sure if a list item applies to your research, read the appropriate section before selecting a response.

Materials & experimental systems

n/a	Involved in the study	
☐	☒	Antibodies
☒	☐	Eukaryotic cell lines
☒	☐	Palaeontology and archaeology
☐	☒	Animals and other organisms
☒	☐	Human research participants
☒	☐	Clinical data
☒	☐	Dual use research of concern

Methods

n/a	Involved in the study	
☒	☐	ChIP-seq
☒	☐	Flow cytometry
☒	☐	MRI-based neuroimaging

Antibodies

Antibodies used	We paid Pacific Immunology to raise antibodies against the Cry14Ab protein, and used the antibodies for western blots to detect the protein in transgenic plants.
Validation	The antibody was tested for sensitivity and specificity using plant tissue, and Cry14Ab protein purified from bacterial expression.

Animals and other organisms

Policy information about studies involving animals; ARRIVE guidelines recommended for reporting animal research

Laboratory animals	Caenorhabiditis elegans strain N2 was used at all stages of the life cycle. Heterodera glycines strain OP50 and field populations were used at all stages of the life cycle.
Wild animals	Field populations of Heterodera glycines were used at all stages of the life cycle. Samples were destroyed by autoclaving after analysis.
Field-collected samples	Field populations of Heterodera glycines were used at all stages of the life cycle. Samples were destroyed by autoclaving after analysis.

Ethics oversight	No ethical approval or guidance was required for working with the nematodes Caenorhabditis elegans and Heterodera glycines.

Note that full information on the approval of the study protocol must also be provided in the manuscript.
